# Improvement of the Space Charge Suppression and Hydrophobicity Property of Cellulose Insulation Pressboard by Surface Sputtering a ZnO/PTFE Functional Film

**DOI:** 10.3390/polym11101610

**Published:** 2019-10-03

**Authors:** Yanqing Li, Jian Hao, Jinfeng Zhang, Wei Hou, Cong Liu, Ruijin Liao

**Affiliations:** 1 State Key Laboratory of Power Transmission Equipment & System Security and New Technology, Chongqing University, Chongqing 400044, China; starwade@163.com (Y.L.); cquhouwei@163.com (W.H.); cqu_lc@163.com (C.L.); rjliao@cqu.edu.cn (R.L.); 2 Key Laboratory of Engineering Dielectrics and Its Application, Ministry of Education, Harbin University of Science and Technology, Harbin 150080, China; zhangjinfeng_phd16@hrbust.edu.cn

**Keywords:** nano-structure functional film, magnetron sputtering, cellulose insulation polymer, space charge, hydrophobicity, zinc oxide, polytetrafluoroethylene

## Abstract

Oil-impregnated cellulose insulation polymer (oil-paper/pressboard insulation) has been widely used in power transformers. Establishing effective ways of improving the physical and chemical properties of the cellulose insulation polymer is currently a popular research topic. In order to improve the charge injection inhibition and hydrophobic properties of the cellulose insulation polymer used in power transformers, nano-structure zinc oxide (ZnO) and polytetrafluoroethylene (PTFE) films were fabricated on a cellulose insulation pressboard surface via reactive radio frequency (RF) magnetron sputtering. Before the fabrication of their composite film, Accelrys Materials Studio (MS) software was applied to simulate the interaction between the nanoparticles and cellulose molecules to determine the depositing sequence. Simulation results show that the ZnO nanoparticle has a better adhesion strength with cellulose molecules than the PTFE nanoparticle, so ZnO film should be sputtered at first to fabricate the ZnO/PTFE composite film for better film quality. The sputtered, thin films were characterized by X-ray photoelectron spectroscopy (XPS), scanning electron microscopy (SEM), and X-ray diffraction (XRD). The space charge injection behavior and the hydrophobicity performance of the untreated pressboard; and the cellulose insulation pressboard with sputtered nano-structure ZnO, PTFE, and the ZnO/PTFE functional films were compared with each other. X-ray photoelectron spectroscopy results showed that ZnO, PTFE, and ZnO/PTFE functional films were all successfully fabricated on the cellulose insulation pressboard surface. Scanning electron microscopy and XRD results present the nano-structure of the sputtered ZnO, PTFE, and ZnO/PTFE functional films and their amorphous states, respectively. The ZnO/PTFE composite functional film shows an apparent space charge suppression effect and hydrophobicity. The amount of the accumulated space charge in the pressboard sputtered ZnO/PTFE composite functional film decreased by about 40% compared with that in untreated cellulose insulation pressboard, and the water contact angle (WCA) increased from 0° to 116°.

## 1. Introduction

High voltage direct current (HVDC) power systems have been utilized in long-distance power energy transmission. The most important apparatus in HVDC systems is the HVDC converter transformer. The converter transformer mainly consists of a steel core, and winding and insulating materials. The insulation materials are mainly the combination of oil and cellulose insulation polymers (cellulose insulation paper/pressboard). The oil-paper/pressboard insulation in the valve winding and outlet bushing of the HVDC converter transformer, simultaneously experience AC, DC, and transient impulse voltages under operation [1]. Under the voltage of DC component, space charge accumulation within the solid insulation material has been regarded as a major issue affecting the safe and reliable operation of the converter transformer [1]. The formation of space charge results in a distortion of the electric field distribution and may lead to local electric field enhancement, thus further inducing aging, partial discharge, and even breakdown, which could ultimately bring about insulation failure [2,3,4]. Therefore, effective methods to suppress space charge accumulation in the oil-paper insulation have always been the focus of research in recent years.

Moreover, moisture is regarded as “the first enemy” after temperature [5,6], which can reduce the thermal life and electrical breakdown strength of the oil-paper insulation. Water is produced during the decomposition of the cellulose. The water produced undergoes a migration from cellulose to oil and vice versa. The hygroscopicity of the cellulose and the water solubility in oil determines the equilibrium of water migration. Water can accelerate the decomposition of the cellulose, which means the more water content in the insulation paper, the faster the thermal aging process. Water content in oil can also dramtically lower the dielectric strength of oil when exceeding the saturation limit. In addition, the appearance of water at the interface of cellulose and oil may lead to partial discharges on the surface [7]. Field experience shows that the moisture content of the oil-impregnated insulation paper in a transformer is usually below 0.5% in the initial stage of operation; however, it may increase to 2%–4% at the terminal stage of its lifetime [6]. Therefore, improving the hydrophobicity of insulating paper is conducive to a partial suppression effect on water migration, thus ensuring reliable insulation performance.

As mentioned above, research on finding effective ways to improve the space charge accumulation phenomenon and hydrophobicity of the pressboard is of great significance. The introduction of nanomaterials is considered to be a popular way of modifying materials. The nanocomposite was first reported by Lewis in 1994 [8], and since then, nano-modification has become a popular way to improve the performance of insulating materials especially polymers. It was demonstrated by some researches that the bulk doping of different nano-particles could improve some properties of dielectric materials. For the cellulose insulation paper, the electrical properties of nano-Al_2_O_3_ and nano-SiO_2_ doped paper are better than those of conventional paper. In addition, there is less space charge accumulation in the bulk of the modified paper [9,10]. Researches on nano-doping in other insulating materials, such as low-density polyethylene (LDPE) and epoxy resin, also showed a dramatic effect of doping nanoparticles on the suppression in space charge accumulation [11,12,13,14]. Nevertheless, the positive effect of nano-doping on the materials is limited by the aggregation of nanoparticles, due to their high surface energy. Besides bulk doping, another way to utilize the nano-effect is surface treatment. The surface modification of materials has gained enormous importance due to the ability to controllably change physical and chemical properties of solid surfaces without affecting the bulk properties [15]. Milliere et al. managed to mitigate the charge injection from an electrode into LDPE by magnetron sputtering a polymer composite layer containing silver nanoparticles [16]. However, rarely does research go into achieving the space charge suppression of the insulation pressboard by surface treatment. From the above, it is worth investigating the fabrication of a special functional nano-structure film on the surface of the cellulose pressboard, which could inhibit space charge accumulation and simultaneously make the cellulose polymers hydrophobic.

Zinc oxide is a wide band gap (3.4 eV) transparent oxide semiconductor material, with the advantages of high electron mobility and visible light transparency [17]. In recent years, ZnO has been attracting more and more attention. Nano-ZnO is believed to be beneficial to charge transport [18]. Moreover, fluorocarbon polymer-like films have been utilized in low friction coatings, excellent dielectric films, and optical coatings [19,20]. Polytetrafluoroethylene also has excellent insulation and hydrophobicity performance [19,20]. It can be imagined that if the coated functional film can have both the advantages of ZnO and PTFE somehow, it will play a role in improving the performance of the cellulose insulation pressboard.

In this paper, first, the ZnO, PTFE, and ZnO/PTFE functional films were each deposited on the surface of the cellulose insulation pressboard by radio frequency (RF) magnetron sputtering. Then, the physical and chemical characteristics of the as-prepared functional films were analyzed. Finally, we investigated the influence of the deposited functional films on the space charge behavior and the hydrophobicity of the cellulose insulation pressboard.

## 2. Experiments

### 2.1. Material Studio Simulation

**Figure 1:**
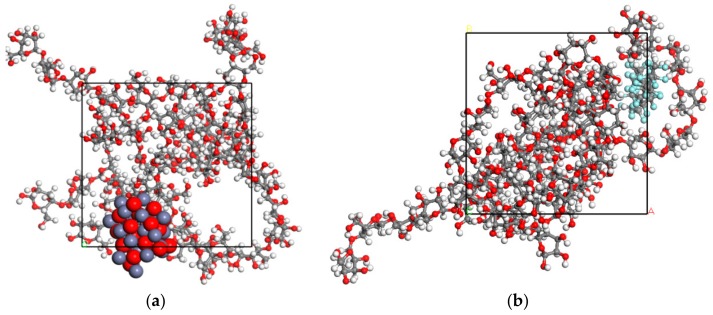
The initial models of (**a**) a ZnO nanoparticle and cellulose, and (**b**) a PTFE nanoparticle and cellulose.

**Figure 2:**
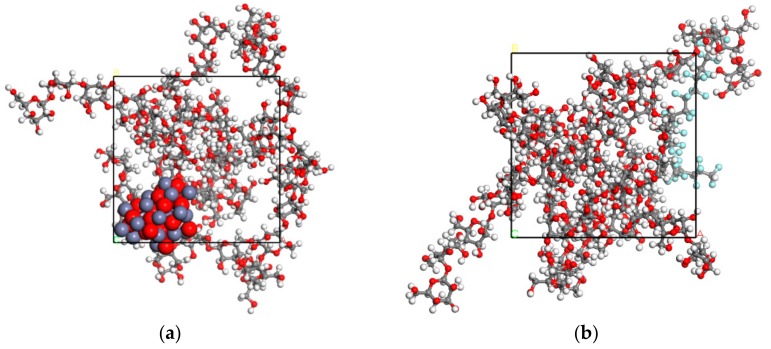
The optimized models of (**a**) a ZnO nanoparticle and cellulose, and (**b**) a PTFE nanoparticle and cellulose.

The optimal depositing sequence should be determined before the fabrication of the composite film on the insulation pressboard surface. We believe that a better film adherence contributes to better film quality. From this perspective, we decided to calculate the interactions bewteen different nanoparticles and cellulose molecules with the help of Accelrys Materials Studio (MS) software (BIOVIA, San Diego, CA, USA). All simulations were carried out in the Forcite and Amorphous Cell modules included in the MS software. The amorphous region of the insulation pressboard was built following a method proposed by Theodorou et al [21]. Previous simulation results show that the length of cellulose chains has little influence on the molecular conformation and physico-chemical properties [22]. Therefore, in this simulation, the cellulose chains with 3, 4 and 5 degrees of polymerization (DP) were used to establish amorphous region models of the pressboard—each being comprised of two cellulose chains, which thereby took the interactions between cellulose chains into consideration. In addition, the radius of the ZnO and PTFE nanoparticle was set at 5 Å according to reference [23]. To make the built model reasonable, geometry and energy optimization was required [24]. The treatment process contained structural refinement, volume relaxation, and annealing. In the structural refinement and volume relaxation process, the default Smart algorithm was applied, which meant a rough optimization by the ‘Steepest descent’ method followed by a further optimization in conjugate gradient method with 10,000 steps. Then, in the annealing treatment, the temperature started from 300 to 650 K and then dropped in the increment of 43.75 K until the initial state 300 K was reached under a canonical ensemble (i.e., NVT ensemble, in which the values of particle number N, volume P, and Temperature T are fixed). The annealing time was set at 100 ps and energy minization was carried out for every annealing step. After the above treatment, the optimized models used for the next simulation in molecular dynamics were built. Figure 1 and Figure 2 show the initial and optimized models in a and b, respectively.

In the next stage, a molecular dynamics (MD) simulation was performed in the NVT ensemble. The nose temperature control method was used and the pressure was set to standard atmospheric pressure by using the Berendsen control method [25]. The force field adopted was a COMPASS II force field, which was a high-quality molecular force field that integrated organic and inorganic molecule parameters into the same force field. The time of MD simulation process was set at 500 ps, and the dynamics information of each atom in the system was collected once every 1000 fs.

### 2.2. Sample Preparation

**Figure 3:**
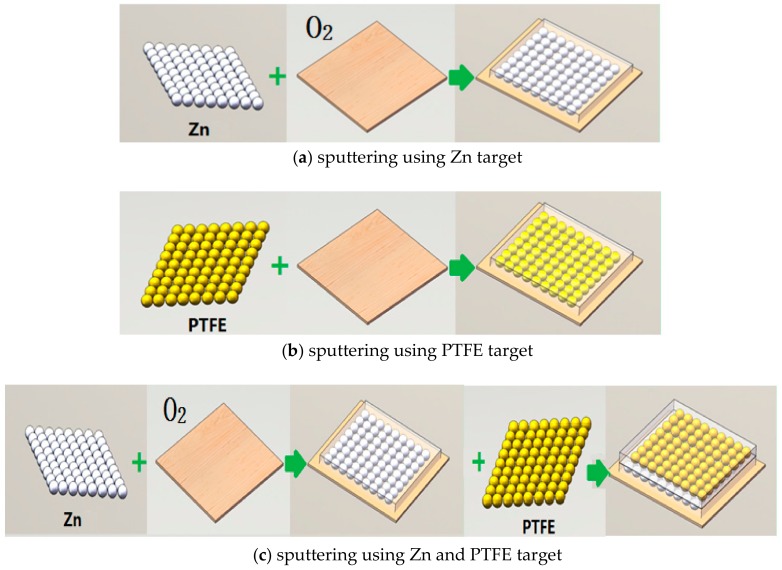
Radio frequency (RF) magnetron sputtering functional film on the surface of cellulose pressboard.

The cellulose insulation pressboard (thickness 0.5 mm) was provided by the NARI Borui transformer factory, Chongqing, China. The insulation pressboard was cut into the size of 15 cm × 10 cm and then used in the RF magnetron sputtering experiment. The JPGF-480 RF magnetron sputtering device (Beijing Instrument Factory, Beijing, China) at 13.56 mHZ was used in this experiment. The cellulose insulation pressboard surface was initially deposited by RF magnetron sputtering of Zn target and PTFE target separately. The Zn and PTFE targets were provided by Zhongnuo XinCai Company, Beijing, China. The diameter was 61.5 mm, the thickness was 6 mm, and the purity was 99.999% for both targets. For the Zn target, sputtering was conducted in argon (Chongqing Hong Hao Gas Co., Ltd., Chongqing, China) plasma under a working pressure of 1.5 Pa and a constant sputtering power at 100 W with a fixed target-substrate distance of 10 cm. Oxygen (Chongqing Hong Hao Gas Co., Ltd., Chongqing, China) was used as the reactive gas at a flow of 20 sccm. The deposition time was 10 min for a better film performance according to multiple trials and to reference [26]. For the PTFE target, the cellulose insulation pressboard surface was sputtered for 20 min (1.5 Pa, 100 W), without reactive gas. To deposit the composited functional film, the cellulose pressboard surface was sputtered by Zn target for 10 min, and then sputtered by PTFE target for 20 min. The deposition parameter was the same as above. Figure 3 is the schematic diagram for the sample sputtering, and abbreviations for each sample are listed in Table 1.

### 2.3. Characterization Method

Characterization of chemical composition was performed by XPS (Thermo Escalab 250 Xi, Waltham, MA, USA) with Al Kα X-rays source. The surface topographies and morphologies of cellulose polymer and sputtered films were analyzed through field emission scanning electron microscope (FE-SEM; JEOL JSM-7800F, Tokyo, Japan). In addition, XRD (PANalytical Empyrea, Almelo, the Netherlands) was performed to analyze the crystalline structure of the deposited film. 

**Figure 4:**
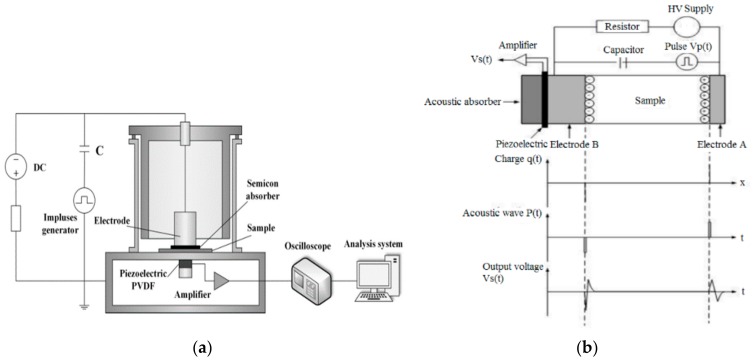
Schematic principle PEA method [27]: (**a**) experiment setup; (**b**) pulsed electro-acoustic (PEA) principle.

Besides the characterization on the structural property, the space charge suppression effect and the contact angle with water were also studied. Researchers have used the pulsed electro-acoustic (PEA) method to measure the space charge in solid dielectrics. The principle of the PEA method can be seen in many studies [4,27]. In brief, it consists of detecting the acoustic waves generated by internal charges under the Coulomb force of a pulsed electric field. The waves are detected by an external piezoelectric transducer, which converts the acoustic signal into an electrical signal. Then, the internal charge density is deduced by signal processing and mathematic treatment. The PEA principle is schematically represented in Figure 4 [27], where q(t) is the electric charge distributed in the sample, P(t) is the acoustic pressure wave as a function of time, the shape of P(t) is the same as the pulse electric field, and Vs(t) is the transducer output as a voltage signal. The PEA system (Shanghai Jiaotong University, Shanghai, China) has a pulse voltage of 600 V and a width of 5 ns. The bottom electrode is made of a 10 mm thick aluminium plate, and the top electrode is the semiconducting polymer film.

Before the space charge measurement, the sputtered pressboard was cut to 30 mm × 30 mm and dried in the vacuum chamber under 50 Pa/90 °C for 24 h. The moisture content of the samples was less than 1%, and technical function of the samples fulfilled the standard IEC 60641-3-1:2008. Meanwhile, new transformer mineral oil was treated by oil filter to remove gas, moisture, and impurities, and also dried under 50 Pa/105 °C for 24h. Moisture content of the oil was less than 10 ppm and the electrical property of the oil fulfilled the standard IEC 60296-2012. The insulation pressboard samples and oil were sealed into bottles and set under 50 Pa/40 for 48 hours to make the insulation pressboard immersed sufficiently. In this test, the applied direct current (DC) electric field was 15kV/mm, and the voltage-on time was 30 min.

Finally, the static water contact angle (WCA) was measured with a Kyowa contact angle meter (Kyowa Electronic Instruments Co., Ltd., Tokyo, Japan) by dispensing 8 µL distilled deionized water droplets. Three different spots for one sample were measured each time, and the average value was regarded as the contact angle and used for subsequent analysis.

## 3. Results and Discussion

### 3.1. Molecular Dynamics Simulation Results

**Figure 5:**
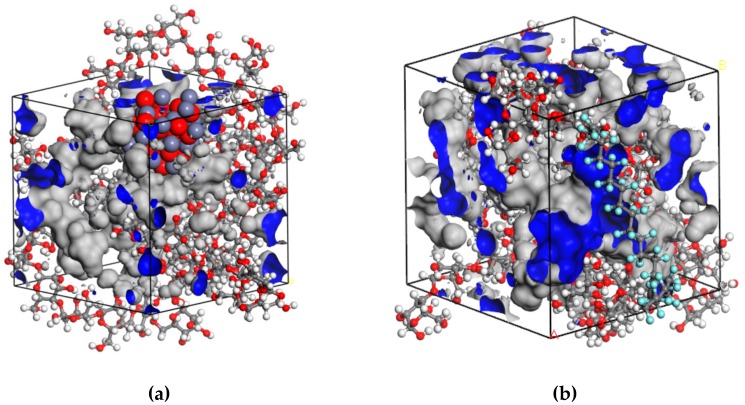
RF magnetron sputtering functional film on the surface of the cellulose pressboard.

Figure 5 demonstrates some of the molecular dynamics simulation results by giving the detail of cell volume in ZnO-cellulose and PTFE-cellulose system. The grey part shows the occupied volume and the blue one stands for free volume. The occupied volumes for the ZnO-cellulose and PTFE-cellulose systems were 8409.06 Å^3^ and 8421.44 Å^3^, while the free volumes were 1620.01 Å^3^ and 2440.65 Å^3^ respectively. The introduction of fractional free volume (FFV) is to measure the void between the molecules inside the material which gives the molecular chains spaces to move. We can calculate the FFV based on the Equation (2) when the temperature is below glass transition temperature. The FFV was 16.15% for ZnO-cellulose system and 22.5% for PTFE-cellulose system, which means there are more free spaces for the motion of molecules in PTFE-cellulose system. This could be attributed to the ZnO nanoparticle’s stronger adherence with cellulose molecules than the PTFE nanoparticle.

(1) $$\mathrm{FFV}=\frac{Volume_{free}}{Volume_{free}+Volume_{occupied}}$$

The interaction between nanoparticles and cellulose molecules could be quantified by the energy calculated during the molecular dynamics simulation. In the ZnO-cellulose model, we calculated the total energy of the system, which was –20854.7 kcal/mol. Then, the energies of ZnO and cellulose were calculated respectively, by getting rid of the other molecules. The total energy of cellulose molecules was calculated to be 1995.8 kcal/mol, and the figure for ZnO nanoparticle was –22506.4 kcal/mol. The energy value does not have a practical meaning because the standard zero value was set randomly during the simulation. However, the difference between the sum of energy of these two materials and the total energy of the system does mean a lot. Based on the Equation (2), we can calculate the interaction energy of ZnO nanoparticle and cellulose molecules. The result turns out to be 344.1 kcal/mol. For another PTFE-cellulose model, the same calculation method was applied and the interaction energy between PTFE nanoparticle and cellulose molecules is 77.6 kcal/mol. We can see that the interaction energy of ZnO and cellulose is higher than PTFE and cellulose. As a result, the sputtered ZnO film will have a better adhesion strength than PTFE film. This indicates that sputtering ZnO film first is the optimal choice. Moreover, the physici-chemical properties of PTFE are so stable as to enjure the acid, alkali, and some other extreme environments. In other words, PTFE film may act as a protective layer on the surface. The sputtering sequence is also reasonable in this form.

(2) $$∆E=\left(E_{1}+E_{2}\right)-E_{total}$$

### 3.2. Chemical Composition Analysis

**Figure 6:**
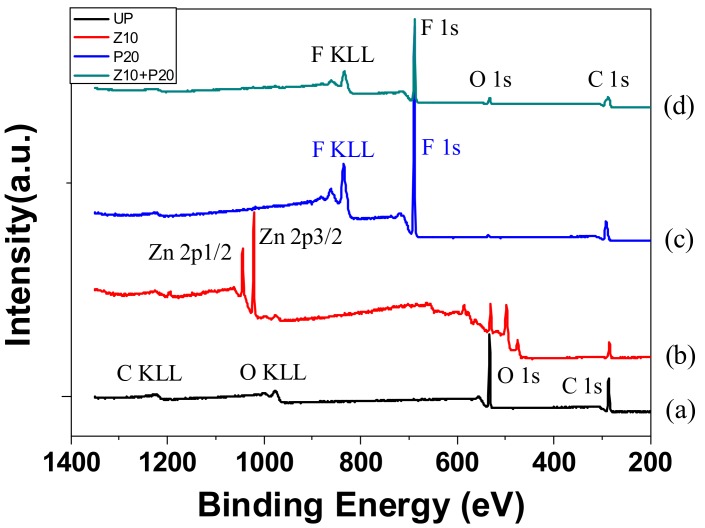
The X-ray photoelectron spectrpmetry (XPS) spectra of (**a**) UP, (**b**) Z10, (**c**) P20, and (**d**) Z10+P20.

Figure 6 presents a comparison of the XPS survey spectra of the untreated pressboard (UP) and the sputtered pressboard. Cellulose insulation pressboard consists of linear, polymeric chains of cyclic, β-d-glucopyranose units, which are composed of C, H, and O elements [28]. There was only the C_1s_ peak, the O_1s,_ peak, and the auger peak of C and O in UP, as labeled in Figure 6. After sputtering, new peaks of Zn atoms and F atoms appeared for sample Z10 and sample P20, respectively. As for sample Z10+P20, only peaks of F atoms were observed in the survey XPS spectra. The peak height of F_1s_ was 40,123, whereas that of Zn_2p_ was 2053. The reason is that the film containing zinc oxide is that is was beneath the fluorocarbon film. Therefore, the peaks of Zn_2p_ atoms are so weak as to be invisible in Figure 6. The above results indicate that the zinc oxide and fluorocarbon film has been fabricated on the pressboard surface.

**Figure 7:**
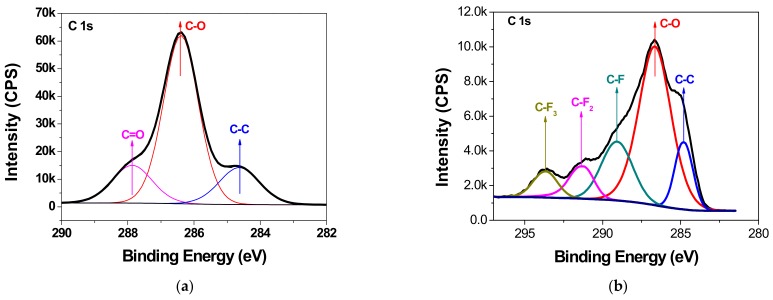

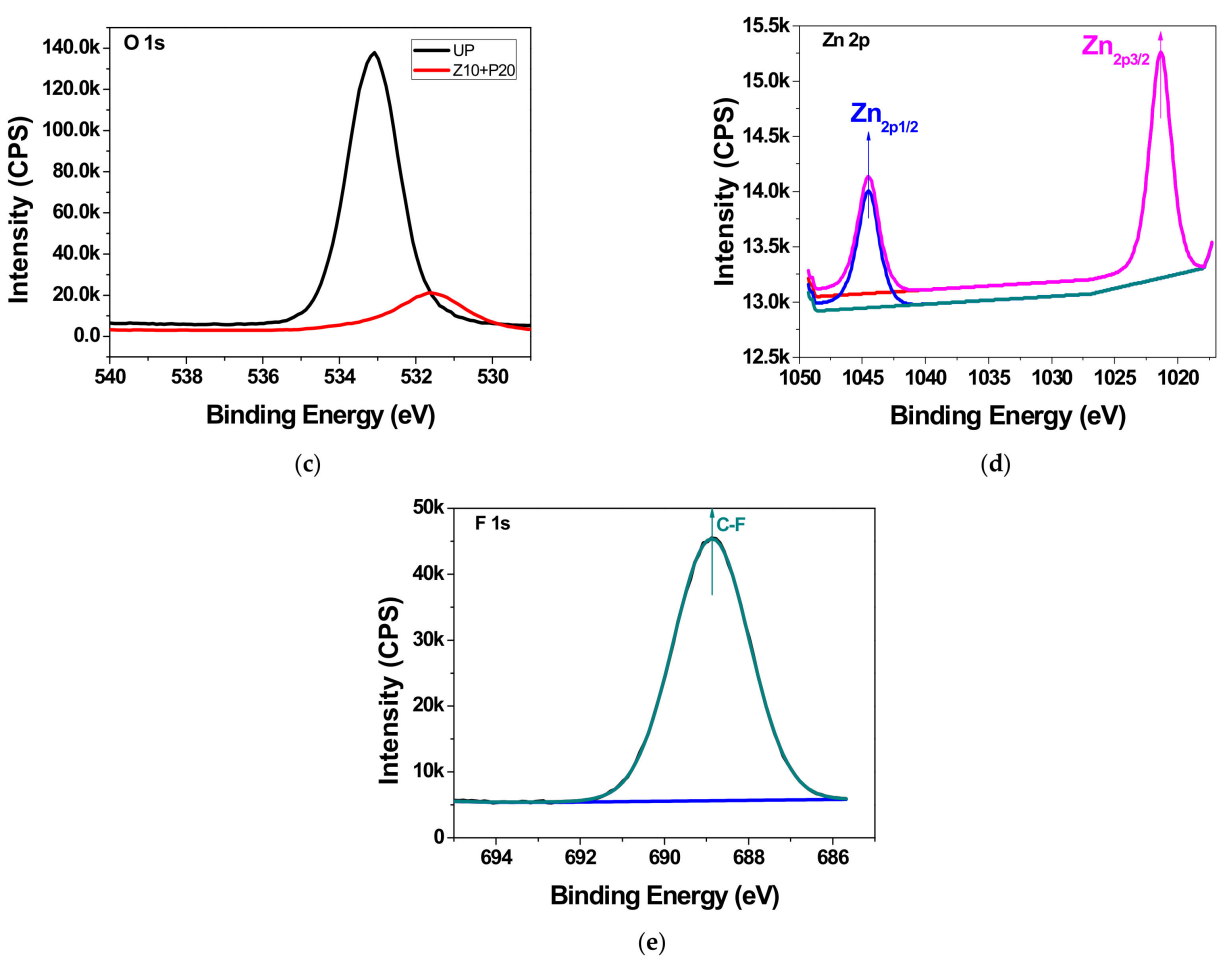
XPS resolution spectra of (**a**) C_1s_ for UP, (**b**) C_1s_ for Z10+P20, (**c**) O_1s_ for UP and Z10+P20, (**d**) Zn_2p_ for Z10+P20, and (**e**) F_1s_ for Z10+P20.

The existence of chemical bonds sputtered on the cellulose polymer surface is determined by high-resolution XPS spectra. Figure 7a,b show the narrow scan spectra results of UP and Z10+P20, respectively. In Figure 7a, the peak at 284.6 eV corresponds to carbon-carbon (C–C) bond or carbon-hydrogen (C–H) bond; the peak at 286.4 eV is due to bonding of carbon to a single non-carbonyl oxygen (C–O); and the peak at 287.9 eV represents bonding of one carbonyl oxygen to a carbon atom (C=O). These three peaks are consistent with the results for cellulose [28]. After the functional film deposition, high-resolution scans revealed C_1s_ spectra containing peaks at 289.08 eV, 291.28 eV, and 293.68 eV, corresponding to C–F, C–F_2_, and C–F_3_ bonds, respectively. The O_1s_ peak in Figure 7c confirms the C–O bond at 533.08 eV on the surface of UP. After sputtering, the O_1s_ peak position shifted to lower binding energy at 531.58 eV. The binding energy of ZnO in O_1S_ spectra stands at 531.1 eV. This results of peak shifting are consistent with oxygen binding mainly to zinc. In addition, the O_1s_‘s peak height decreased sharply after sputtering due to the formation of film on the cellulose surface; thus, little information from cellulose was detected, and the zinc content was not very abundant because of the short sputtering time (10 min). In Figure 7d, two peaks appear at 1021.8 eV for Zn_2p3/2_ and 1044.8 eV for Zn_2p1/2_, confirming the formation of Zn–O bond. In addition, there is no peak located at around 88 eV corresponding to a Zn–Zn bond. The results demonstrate that the zinc was completely oxidized during the reactive RF magnetic sputtering treatment using O_2_. Figure 7e shows the F_1s_ spectral result. There is also a new peak located at 688.88 eV corresponding to a C–F bond after sputtering. From the above, it could be concluded that the ZnO/PTFE film was successfully fabricated on the cellulose insulation pressboard surface.

### 3.3. Surface Morphology

**Figure 8:**
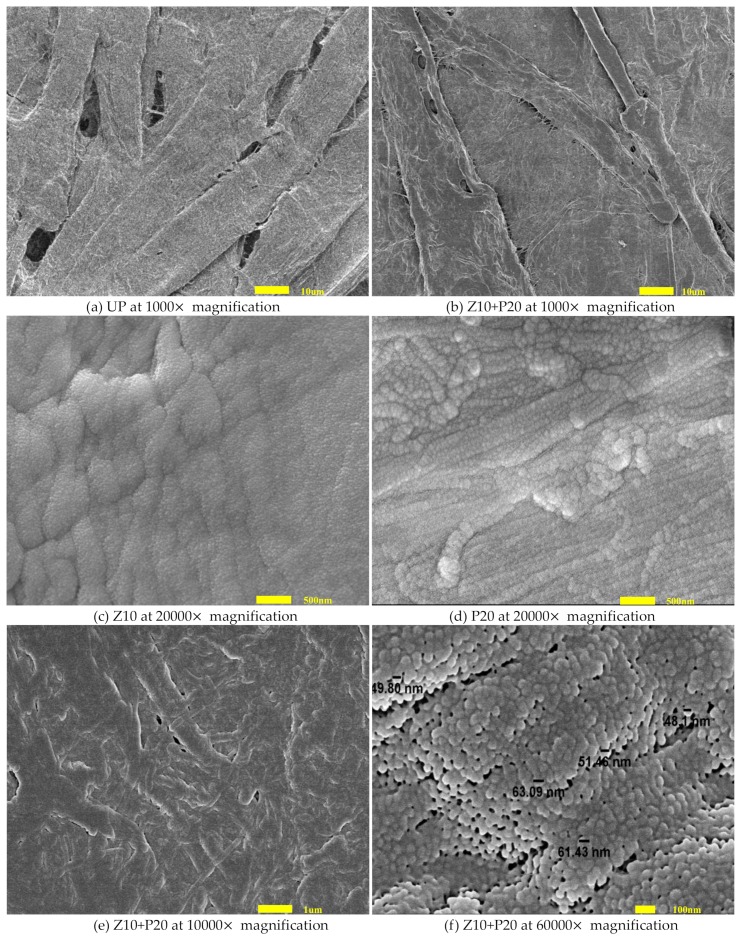
SEM images of (**a**) UP, (**b**) Z10+P20, (**c**) Z10, (**d**) Z10+P20, (**e**) Z10+P20, and (**f**) Z10+P20.

Scanning electron microscopy images of the untreated pressboard and the sputtered pressboard surface are illustrated in Figure 8. It can be observed that the fibers of UP (Figure 8a) intersecting each other and the surface were relatively rough. There were some cracks where the fibers intersect. The characteristic structure of the untreated pressboard was highly porous and laminar. The surface of sputtered pressboard (Z10+P20) was smoother and denser, as shown in Figure 8b. To better illustrate the surface morphology of sample Z10+P20, it was necessary to study the morphology of samples Z10 and P20. As presented in Figure 8c,d, the film was comprised of nano-particles with dozens of nanometers in diameter. To be specific, ZnO nanoparticles were small and well isolated from each other. In addition, the distribution of ZnO nano-particles was more uniform and denser than that of PTFE. For P20, the average size of PTFE nano-particles was larger than ZnO nano-particles, and the PTFE nano-particles tended to be in alignment. Scanning electron microscopy images for Z10+P20 at 10,000× magnification (Figure 8e) indicat that an obvious change occurs on the surface. The porosity decreases and individual fibers become harder to identify. With even higher magnification, at 60,000×, there is a dense and uniform distribution of tightly arranged particles deposited on the surface (Figure 8f). It can be seen that the sputtered film is comprised of nano-particles ranging from 40 to 70 nm in diameter. Some nano-particles are clearly seen, whereas some nano-particles are agglomerated. It could be inferred that the newly sputtered particles continually attach and aggregat to the previously deposited particles. The introduction of the nano-particles growing on the fibers could affect the material properties.

### 3.4. Crystalline Structure

**Figure 9:**
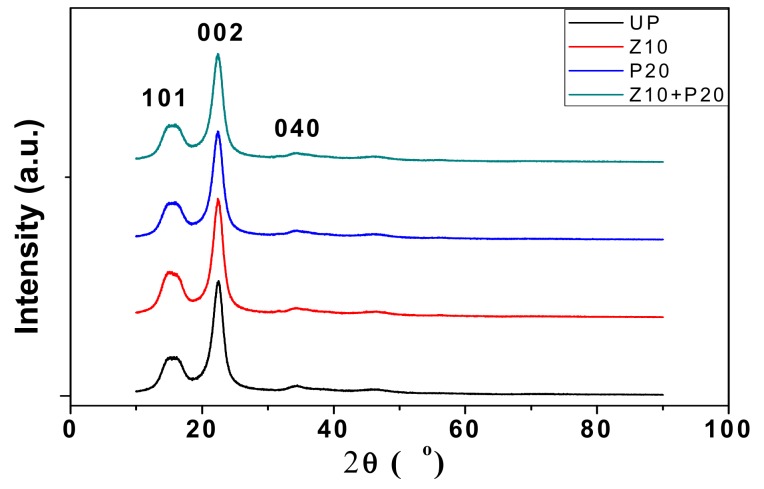
The results of small-angle X-ray diffraction.

X-ray diffraction analysis is very helpful for investigating the crystal structure of the material. Considering that the film sputtered on the surface, the small-angle X-ray diffraction (SAXD) method was used for precise characterization. The gracing angle was set at 1.5°, and the scanning step was 0.02°. Results for the samples—UP, Zn10, P20, and Zn10+P20—are given in Figure 9. The obvious diffraction peaks at 2θ = 14.93°, 2θ = 22.60° and 2θ = 34.85° are the characteristic phase (101), (002), and (040) diffraction peaks of cellulose I, respectively [29]. There are sharp peaks and some dispersive diffraction peaks, which means that the cellulose had a mixed structure of a crystallized and amorphous phase. However, there was no obvious difference between the untreated pressboard and the sputtered pressboard in this experiment. It could be inferred that the film is still on the growth stage when the sputtering time is short, thus ZnO did not show any specific crystal structure. Furthermore, the PTFE film also existed in an amorphous form on the surface of insulation pressboard.

### 3.5. Space Charge Suppression Effect

The threshold electric field for space charge in oil-impregnated insulation paper is usually about 10–12 kV/mm [30]. When the applied electric field is higher than 10–12 kV/mm, charge injection from the metal electrode to the pressboard through the contact is inevitable due to the Schottky injection mechanism and the tunneling effect [2]. Injected charges undergo several processes, such as migration, trapping, detrapping, and neutralization. Charges will keep migrating until these four processes reach an equilibrium. Charge accumulation occurs when the injection process prevails over the charge migrating process due to the high electric field. As mentioned in the introduction, nano-ZnO is believed to speed up the charge migration rate, which could reduce the space charge accumulation. Therefore, in this paper, ZnO nanoparticles are mainly used for the inhibition of space charge accumulation. To confirm the restraining function of the sputtered ZnO film on charge accumulation, the space charge distribution of the untreated and sputtered pressboard under DC electric field 15 kV/mm for 30 min was measured.

**Figure 10:**
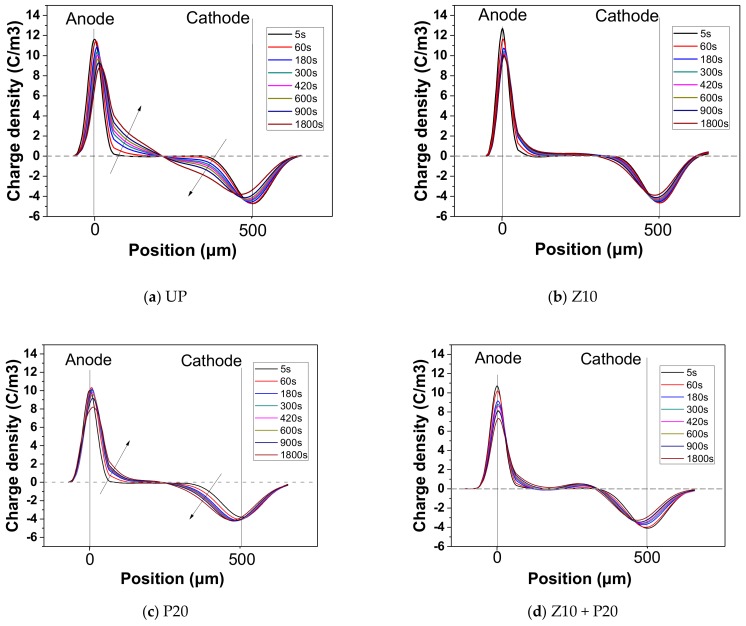
The space charge behavior of (**a**) UP, (**b**) Z10, (**c**) P20, and (**d**) SP under DC 15kV/mm.

In Figure 10a, the anode charge density peak decreases from 11.6 C/m^3^ at 5 s to 8.3 C/m^3^ at 1800 s, whereas the cathode charge density peak decreases from –4.7 C/m^3^ at 5 s to –3.8 C/m^3^ at 1800 s. The charge density on the electrode decreases with the voltage applied time, indicating that space charge moves away from the electrodes into the inner part of the sample. It can be seen in Figure 10b that the anode charge density peak decreases from 12.6 C/m^3^ at 5 s to 9.2 C/m^3^ at 1800 s. It is clear that the space charge accumulated within sample Z10 bulk is obviously less than that in UP. Around the middle position within the sample bulk, the space charge density is around zero value. That is to say, no obvious charge accumulates here. The sample P20 was also tested. As shown in Figure 10c, a similar situation of charge injection occurs, and the charge injection from both anode and cathode takes place, though the trapped charge amount is less compared with UP (Figure 10a). This means the nano-structure PTFE film also seems to have a positive impact on charge accumulation suppression, albeit to a smaller degree. Figure 10d shows the result of sample Z10+P20. It can be seen from the charge distribution that the inhibition effect of space charge is almost the same as the result shown in Figure 10b for Z10. From the comparison we can see that the PTFE film does not weaken the effect of ZnO nano-particles on inhibiting the space charge injection.

The total absolute amount of space charge during the voltage-on period can be calculated using Equation (3), where L is the thickness of the sample,$\;\rho \left(x,\;t\right)\;$ is the charge density at position x, t is the DC voltage applied time, and S is the area of the electrode.

(3) $$Q\left(t\right)=\int_{0}^{L}\left|\rho \left(x,t\right)\right|\mathrm{Sdx}$$

**Figure 11:**
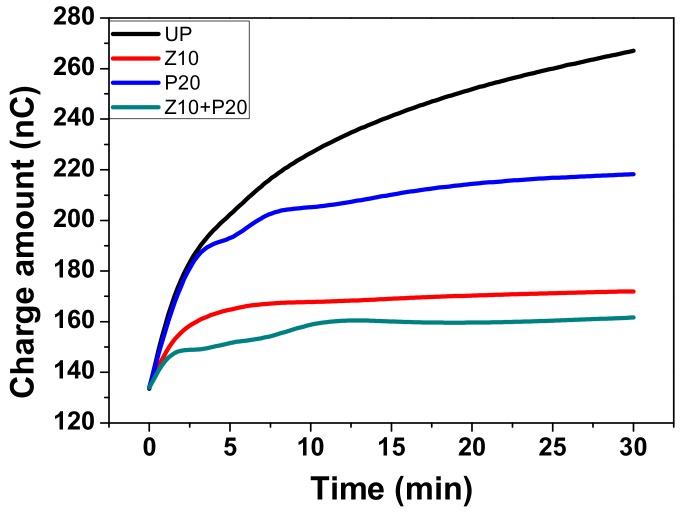
Absolute charge amount in the treated and untreated pressboard.

The number of charges trapped in the bulk of sample UP, Zn10, P20, and Zn10+P20, during the DC electric field 15 kV/mm applied for 0–30 min are shown in Figure 11. The charge amount rises as the voltage-on time increases. It can be clearly seen that the increasing rate of charge amount and the final charge amount of the untreated pressboard are higher than other sputtered pressboards. Specifically, PTFE film made no difference in the initial stage of charge injection, but the charge amount at quasi-equilibrium state is lower than the UP. As for ZnO film, the lower increasing rate of charge amount confirmed that it clearly plays a significant role in charge injection inhibition. Moreover, the composited functional film sample ZnO/PTFE has the best result among all the samples, with both the lowest increasing rate of charge amount and the lowest total charge amount at equilibrium. Compared with untreated cellulose insulation pressboard, the amount of the trapped charges in the pressboard sputtered ZnO/PTFE composited functional film decreases by about 40%. From this perspective, the pressboard sputtered composited functional film ZnO/PTFE presents considerable performance for the space charge inhibition.

To study the impact of space charge accumulation on the electric field distortion, the electric field strength was calculated by Equation (4), where $\;\rho \left(x\right)$ is the charge density, $\varepsilon_{0}$ is the vacuum permittivity, $\varepsilon_{r}$ is the relative permittivity of the sample, and L is the thickness of the sample.

(4) $$E\left(x\right)=\int_{0}^{x}\frac{\rho \left(x\right)}{\varepsilon_{0}\varepsilon_{r}}\mathrm{dx}\;0\leq x\leq L$$

**Figure 12:**
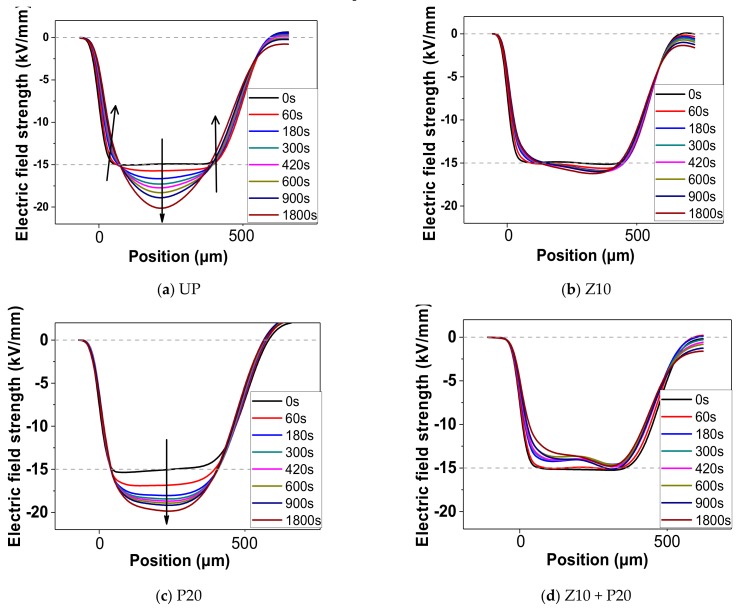
Electric field distribution for different samples: (**a**) UP, (**b**) Z10, (**c**) P20, and (**d**) Z10+P20.

The calculated electric field distribution within each sample is shown in Figure 12. For UP, the injected homo-charge reduces the electric field in the region between electrodes and sample. However, with the charges moving deeper into the bulk, the electric field gradually increases along the abscissa axis and peaks in the middle region of the bulk. Although the applied electric field is 15 kV/mm, the actual maximum electric field reaches 20 kV/mm, which means that the field enhancement, due to the presence of space charge, is around 33%. As for the Z10 sample (Figure 12b), the result shows that there is no obvious electric field distortion due to little space charge accumulation in the bulk, with the highest electric field strength standing at roughly 16 kV/mm. For P20, a weak mitigation effect results in the same obvious electric field distortion as in UP, but the peak value is lower than that in UP. For Z10+P20, the space charge suppression effect is better than Z10, since the actual electric field in the bulk is even lower than the applied electric stress.

### 3.6. Hydrophobicity Analysis

**Figure 13:**
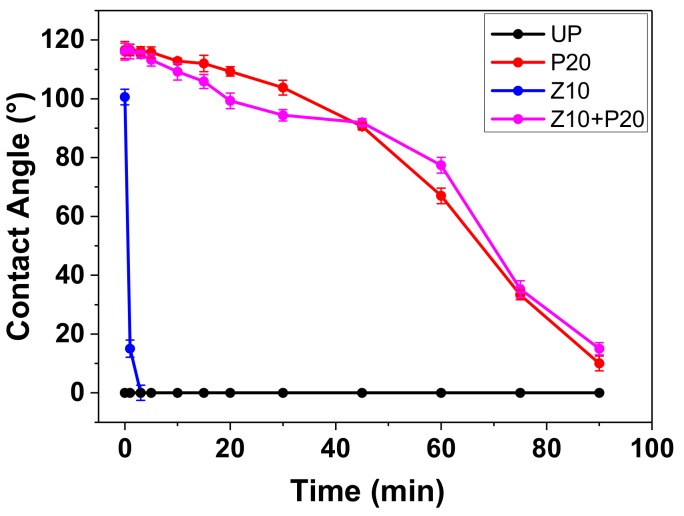
The contact angle of the untreated and sputtered pressboard.

The insulation paper is developed from natural fiber. The structural characteristics of the fiber determine that it absorbs water easily. However, the hygroscopicity of the insulation paper is a very bad feature when used for insulation in a transformer. The moisture content would lead to the decomposition of cellulose and partial discharge. Therefore, if the insulation paper has better hydrophobicity, the negative effect of moisture could be suppressed. Figure 13 shows the contact angle results of water droplets dripping on the surface of untreated and sputtered pressboard.

For UP, the water contact angle was about 0°, indicating that water droplets were absorbed immediately by the pressboard due to the existence of hydroxyl groups in cellulose. For Z10, the water contact angle was high at first but decreased dramatically in few minutes. The surface of the pressboard was coated and the cellulose molecule was surrounded by ZnO nanoparticles, so the hydrophilic effect of the hydroxyl group was weakened. However, as it could be seen in Figure 8b, there were also some defects and cracks in the sputtered pressboard, though less than for UP. Therefore, the water would penetrate and then be absorbed by the cellulose molecules in few minutes. As reported in reference [31], PTFE film has many C–F groups, which show dramatic hydrophobicity. When the pressboard ws sputtered by the PTFE target, the newly formed C-F groups on the surface made the pressboard hydrophobic to some extent, so we can see the contact angle of P20 is 116.6° at the initial stage, as shown in Figure 13. Nevertheless, it could be seen that the contact angle decreased gradually with time, which meant the hydrophobicity of the PTFE film above the cellulose substrate could not endure a quite long time. This phenomenon also resulted from the property of the cellulose substrate, where the effect of hydroxyl group could just be weakened but not eliminated. Thus, the water droplets were absorbed into the substrate eventually. Figure 13 also showed the comparative results of sample Z10+P20 and the P20 sample. The starting value of sample Z10+P20 was almost the same as sample P20. In the beginning, the contact angle of Z10+P20 decreased faster than P20, but it was reversed in the later stage. Overall, the hydrophobicity of P20 and Z10+P20 were almost the same. It could be inferred that the deposited Z10+P20 composited functional film has a lower moisture absorption rate according to reference [32], which is good for insulation pressboard used in power transformers.

### 3.7. Tensile Strength and Electric Breakdown Values

Tensile strength and electric breakdown values are two basic and critical parameters for the insulation pressboard, so we compared these two parameters of the untreated and treated pressboard, and the results were shown in Table 2. The tensile strength of the composite pressboard was directly tested after sputtering according to ISO 1924-2:2008. The samples used for DC and AC breakdown test were prepared the same way as the samples for PEA test, and the test was conducted by plate-plate electrodes according to IEC 60243-1:2013 standard. Every test was performed eight times and the mean value was calculated for Table 2. It is shown that there was no big difference between the untreated and sputtered composite pressboard. The sputtered pressboard also accompolished the standard IEC 60641-3-1:2008.

## 4. Conclusions

The nano-structures ZnO, PTFE, and their composite films are designed to improve the insulation performance of the cellulose pressboard. Results from Materials Studio show that the ZnO nanoparticle has a better adhesion strength with cellulose molecules than the PTFE nanoparticle. Hence, ZnO film should be sputtered first to fabricate the ZnO/PTFE composite film.

Materials Studio simulation results show that ZnO nanoparticle has a better adhesion strength with cellulose molecules than PTFE nanoparticle. The ZnO film should be sputtered at first to fabricate the ZnO/PTFE composite film for better film quality.

X-ray photoelectron spectroscopy analysis shows that the ZnO/PTFE composited functional film was successfully fabricated on the cellulose insulation polymer surface by reactive RF magnetron sputtering. The SEM results show that there were dense and uniform nano-particles ranging from 40 to 70 nm in diameter deposited on the surface of cellulose. The XRD results show that the nano-structure of ZnO and PTFE was of an amorphous form.

The sputtered nano-structure ZnO film on the cellulose polymer surface has an obvious space charge suppression effect, and the sputtered nano-structure PTFE film could turn the cellulose surface from hydrophilicity into hydrophobicity. The nano-structure ZnO/PTFE composited functional film could integrate the advantages of nano-structure ZnO and PTFE film at the same time. The amount of the accumulated space charge in the pressboard sputtered ZnO/PTFE composite functional film decreases by about 40% compared with that in untreated cellulose insulation pressboard, and the water contact angle increases from 0° to 116°.

The tensile strength and electrical breakdown test results showed that the basic properties of the sputtered composite pressboard were not weakened, and achieved the standard ISO 5269-2:2004.

It is concluded that ratio frequency magnetron sputtering is an effective way to enhance the performance of the insulation pressboard in some aspects, without weakening the basic properties. This work provides a method for the development of high-performance cellulose insulation polymer used in HVDC equipment.

## Figures and Tables

**Table 1:** Sample composition.

Sample	Abbreviation
Untreated pressboard	UP
Pressboard sputtered Zn for 10 min (reactive O_2_)	Z10
Pressboard sputtered PTFE for 20 min	P20
Pressboard sputtered Zn for 10 min (reactive O_2_) and PTFE for 20 min	Z10+P20

**Table 2:** Tensile strength and electric breakdown test results.

Sample	Tensile Strength (Mpa)	Standard Value (Mpa)	AC Breakdown (kV/mm)	DC Breakdown (kV/mm)	Standard Value (kV/mm)
UP	49.91	>40	55.28	136.37	>40
Z10+P20	50.48	>40	56.26	144.27	>40
